# Sampling of basement fluids via Circulation Obviation Retrofit Kits (CORKs) for dissolved gases, fluid fixation at the seafloor, and the characterization of organic carbon

**DOI:** 10.1016/j.mex.2020.101033

**Published:** 2020-08-15

**Authors:** Huei-Ting Lin, Chih-Chiang Hsieh, Daniel J. Repeta, Michael S. Rappé

**Affiliations:** a Institute of Oceanography, National Taiwan University, No.1, Sec. 4 Roosevelt Road, Taipei City 10617, Taiwan; b Department of Oceanography, SOEST, University of Hawaii, 1000 Pope Rd., Honolulu, HI 96822, USA; c Department of Marine Chemistry and Geochemistry, Woods Hole Oceanographic Institution, 360 Woods Hole Rd, Woods Hole, MA 02543, USA; d Hawaii Institute of Marine Biology, SOEST, University of Hawaii, P.O. Box 1346, Kaneohe, HI 96744, USA

**Keywords:** GeoMICROBE, Hydrothermal fluid, Crustal fluid, Mobile pumping system, Helium, Methane, Dissolved organic matter, Extraction and preconcentration, Deep subseafloor

## Abstract

The advanced instrumented GeoMICROBE sleds (Cowen et al., 2012) facilitate the collection of hydrothermal fluids and suspended particles in the subseafloor (basaltic) basement through Circulation Obviation Retrofit Kits (CORKs) installed within boreholes of the Integrated Ocean Drilling Program. The main components of the GeoMICROBE can be converted into a mobile pumping system (MPS) that is installed on the front basket of a submersible or remotely-operated-vehicle (ROV). Here, we provide details of a hydrothermal fluid-trap used on the MPS, through which a gastight sampler can withdraw fluids. We also applied the MPS to demonstrate the value of fixing samples at the seafloor in order to determine redox-sensitive dissolved iron concentrations and speciation measurements. To make the best use of the GeoMICROBE sleds, we describe a miniature and mobile version of the GeoMICROBE sled, which permits rapid turn-over and is relatively easy for preparation and operation. Similar to GeoMICROBE sleds, the Mobile GeoMICROBE (MGM) is capable of collecting fluid samples, filtration of suspended particles, and extraction of organics. We validate this approach by demonstrating the seafloor extraction of hydrophobic organics from a large volume (247L) of hydrothermal fluids.

• We describe the design of a hydrothermal fluid-trap for use with a gastight sampler, as well as the use of seafloor fixation, through ROV- or submersible assisted mobile pumping systems.
• We describe the design of a Mobile GeoMICROBE (MGM) that enhances large volume hydrothermal fluid sampling, suspended particle filtration, and organic matter extraction on the seafloor.
• We provide an example of organic matter extracted and characterized from hydrothermal fluids via a MGM.

## Graphical abstract

**Figure:**
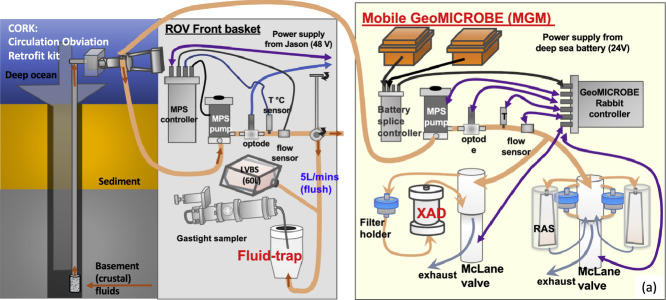

Specifications table

**Table:** 

Subject Area	Earth and Planetary Sciences
More specific subject area	*Oceanography: deep sea research*
Method name	*Hydrothermal-fluid-trap (“fluid-trap”) on advanced mobile pumping system (MPS), and Mobile-GeoMICROBE (MGM) instrumented sled*
Name and reference of original method	*Cowen, J.P., Copson, D.A., Jolly, J., Hsieh, C.-C., Lin, H.-T., Glazer, B.T., Wheat, C.G., 2012. Advanced instrument system for real-time and time-series microbial geochemical sampling of the deep (basaltic) crustal biosphere. Deep-Sea Res. PT I 61, 43-56.*
Resource availability	**Flow sensor with Polyvinylidene fluoride (PVDF) wetted parts:**https://www.btflowmeter.com/en/flow-meter-products/flow-meters-lowflow-flowmeters-low-flow-turbine-flow-meter-fuel-flow-meters-diesel-fuel-flow-meter-watermeters-paddlewheel-flow-meter-oil-flow-meter-oilflowmeter-waterflowmeter-water-meters-turbineflowmeter-oilflowmeter/chemical-flowmeter-extrem-low-flow-pvdf-miniature-size-q-0003-8-lpm.html**Tedlar® polyvinyl fluoride (PVF) bag**s: MiDan Co. 12279 Pipeline Ave.,Chino, CA 91710-2142; Phone: (909) 627-1208,https://www.midanco.com/**Mylar bags**: Jensen Inert Product, 3773 Northwest 126 Ave, Coral Spring, FL, 33065, http://products.jenseninert.com/category/egories-gas-sampling-bags-tedlar-gas-sampling-bags**25-port valve system**: McLane Research Laboratories, Inc.: https://mclanelabs.com/**JACO Fittings**: RYAN HERCO Flow Solutions; Phone: 1-800-848-1141.**Three-way ball valve fitting**: Harrington Industrial Plastics (Hawaii), 91-361 Kaiholo Street Kapolei, Hawaii 96707, U.S.A, Phone: (808) 690-8220**Fitting order information:**JACO fitting: Fittings: https://www.rhfs.com/Request for Kynar material 1. Male compressional tube fitting- MALE CONN 1/4"OD X 1/4"MT PVDF (10-4-4-K-PG) https://www.rhfs.com/root/0602.033 2. Female compressional tube connection-FEM CONN 1/4"OD X 1/4"T PVDF(25-4-4-K-PG) https://www.rhfs.com/root/0607.033. 3. Bulkhead union, 1/4"OD X 1/4"OD PVDF (20-4-K-PG) https://www.rhfs.com/root.html?cdskeys=0617.033 4. Bulk head nut- BKHD NUT 1/4" PVDF, Jaco Manufacturing (K-O-4-B):https://www.rhfs.com/root/0634.603 5. PVDF GRIPPER NUT 1/4" (K-PG-4) https://www.rhfs.com/root/0634.503 6. PVDF washer for 1/4" screw https://www.extreme-bolt.com/products-pvdf-washers.html 7. High-Strength PVDF Film https://www.mcmaster.com/pvdf-film 8. JACO Union Connector, PVDF, 1/4" Tube PVDF JACO 15-4-K-PG 9. MALE CONN 1/2"OD X 1/2"MT PVDF (10-8-8-K-PG) https://www.rhfs.com/root/0602.066 10. Female compressional tube connection-FEM CONN 1/2"OD X 1/2"T PVDF(25-8-8-K-PG) https://www.rhfs.com/root/0607.066

## Introduction

The uppermost 40–500 m of submarine ridge flank basement, with ages between 1 and 65 million-years-old, is fractured and permeable, holds ~2% of the ocean volume and accounts for 70% of the seafloor hydrothermal heat flux [12]. Such a massive power output implies a high flux of volatiles and other thermally and biogeochemically reacted chemical species that require quantification. The ridge-flank basement also has moderate temperatures (<100°C) and hosts a diverse microbial community [4,^15^,20]. Opportunities to study the sedimented ridge-flank basement are provided by Circulation Obviation Retrofit Kit (CORK) observatories installed in selected sealed boreholes drilled by Ocean Drilling Program (ODP; [9]) and Integrated Ocean Drilling Program (IODP, [10]).

In the early deployments, CORKs were designed as “instrumented borehole seal,” and used to provide the opportunity for long-term hydrogeological or seismic monitoring [6]. Later, fluids flowing through a spigot of an over-pressured CORK observatory at borehole 1026B (ODP 168) allowed the collection of suspended particles and organics with a custom-build BioColumn device for probing the sub-basement biosphere [4,5]. However, as fluids flow through the interior of the CORK, they can come in contact with the low alloy steel casing and casing cement, which may impact the chemical and biological integrity of the fluids. In fact, extensive biofilms have been observed on scrapings from the CORK steel after removal from borehole 1026B [21]. After Cowen et al. [4] revealed the existence of a diverse basement community, new advanced designs of CORKs provided samples with reduced contamination suitable for biogeochemistry and biological studies [1,^8^,25]. Critical improvements related to the subbasement microbial biosphere studies include the installation of fluid delivery lines (FDLs) made of stainless steel and Tefzel (i.e., ethylene tetrafluoroethylene, ETFE), intake gratings, and casings adjacent to FDL intake gratings with titanium screens at the fluid intake in the basement [3]. However, the FDLs created significant drag of the fluid flow, and pumping was required to draw fluid through the 260-400 m long pipes.

Seafloor sampling systems were, subsequently, designed, and developed to collect high-quality large-volume basement fluid and suspended particles via CORK FDLs [3]. The advanced GeoMICROBE instrument provides an autonomous fluid and particle sample collection capability in a time-series manner [3]. A standard GeoMICROBE instrumented sled composed of a primary three-to-one solenoid valve system, a positive displacement primary pump, an Optode oxygen sensor, a temperature sensor, a flow sensor, and two McLane 25-port valve manifold systems for fluid sampling and filtration, a custom-build computerized controller (Rabbit Controller), seven 24 V-40 A Deep Sea Power and Light batteries, a battery splice controller, and a wet-mateable (ODI) communication system with submersibles. The custom-built primary pump (named Mega Pump) consists of titanium and Teflon wetted parts manufactured by Micropump®, now a unit of IDEX Corporation, USA. The GeoMICROBE had been deployed for one year along the Juan de Fuca Ridge flank [3] and for 1.5 years at North Pond CORKs in the Atlantic Ocean [20], collecting valuable samples for subbasement microbial and geochemical research. Major components of the GeoMICROBE system, including the pump, sensors, and the controller, were transformed into a mobile pumping system (MPS). The MPS was installed on the front basket of a submersible or a remotely-operated-vehicle (ROV). The versatile MPS system is able to supply crustal fluids into medium volume bag samplers (MVBS; 15L), large volume bag samplers (LVBS; 60L), or direct filtration of fluids, providing samples for microbiological and biogeochemical research [15,16].

In this work, we focus on a modification to the MPS for dissolved gas sampling. Gastight samplers [7] permit precise sampling of hydrothermal fluids for dissolved gas analyses. The gastight is particularly suitable for holding dissolved gas under pressure for up to a month [17,18]. However, the uptake rate of the gastight sampler is >100 mL/s, whereas the fastest fluid delivery rate of our MPS pump is 83 ml/s (5 L/min). Previously, the highest quality gastight sample we were able to collect from a CORK FDL for dissolved gas analyses in 2010 contained only 40% hydrothermal end-member fluid, with background seawater constituting the remaining 60% [16]. A hydrothermal-fluid trap was thus designed to significantly improve the sample quality to 94-100% end-member fluid [17]. Here, we describe the details of the evolving designs of the hydrothermal-fluid-trap that have lead to successful studies of dissolved methane, hydrogen [17], and primordial gas helium-3 (^3^He) [18].

We also describe the application of the MPS to in situ experiments. With the MPS-bag system, even when the sampling bags were pre-evacuated, the plumbing materials allowed trace amounts of oxygen to diffuse into the sampling bags, leading to an increase in dissolved oxygen of up to 4 μM when the sample bags were recovered shipboard (Lin et al., unpublished). This amount of oxygen is sufficient to oxidize reduced compounds such as dissolved iron and dissolved hydrogen sulfide present in the system, leading to difficulty in evaluating the redox status and the energy available for the deep-subsurface microbial biosphere. To circumvent this issue, we demonstrate a mean to pre-charge sample bags with fixatives in order to preserve a reduced compound (in this case, dissolved Fe^2+^) in situ.

Lastly, we provide descriptions on a versatile miniature Mobile GeoMICROBE (MGM) modified from a standard GeoMICROBE, and demonstrate its utility for investigating dissolved organic matter in the deep subseafloor [19]. The MGM minimizes the idle time of a submersible or a ROV on the seafloor to accommodate the slow pump rates for successful filtration and organic matter extraction. Typical filtration and extraction rates were 80–100 mL/min to avoid the backpressure created by the filter and lead to biased results on the recovery of microbial biomass. The minimum sample size for organic characterization is 20 L, which took at least 200 mins. An entire submersible dive of 8 hours only allows the collection of three samples. Thus, the MGM significantly increases our filtration and organic extraction capability.

## Method

### Mobile pumping system (MPS)

#### Overview

**Fig. 1:**
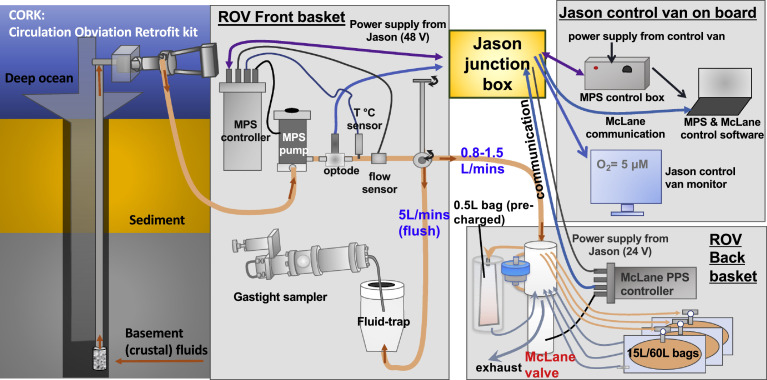
Schematic illustration of flow diagram of the advanced Mobile Pumping System (MPS) coupled to a hydrothermal-fluid-trap on the front basket. A 25-port McLane valve system, a medium volume bag sampler (MVBS), 500 mL pre-chargeable bag, filter holders on the back basket. Purple cable provides power and communication to MPS.

**Fig. 2:**
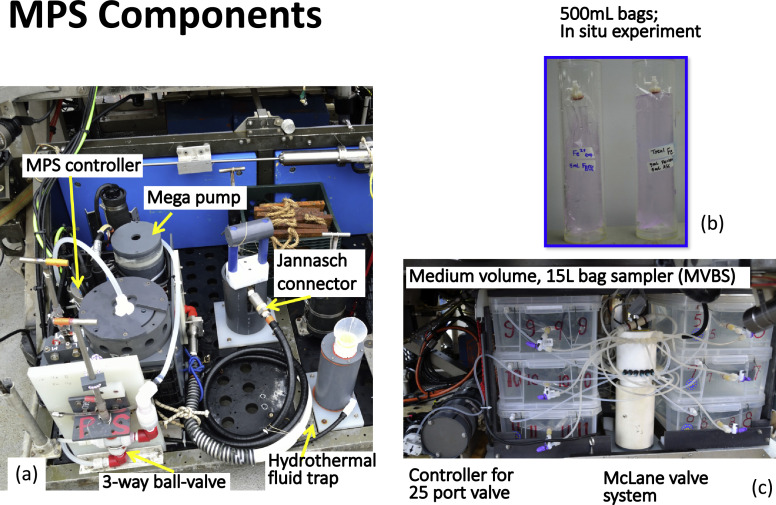
Photos of sampling systems. (a) The mobile pumping system (MPS) on a remotely operated vehicle (ROV). (b) In situ experiment demonstrating the importance of fixative addition for preserving the redox sensitive chemical species. (c) Valve system and medium volume bag sampler (MVBS) located on the back basket of an ROV.

Submarine ridge flank hydrothermal fluids were drawn from the basement through a CORK FDL by the MPS secured on the front basket of the ROV Jason (Figs. 1 and 2). A matching Aeroquip or Jannasch connector [25] connected the MPS to the outlet of the CORK FDL located on the CORK head. The Mega Pump pushed the hydrothermal fluid past an Aanderaa oxygen Optode (Xylem Analytics, U.S.A), an SBE 38 temperature sensor (SEA•BIRD Scientific, U.S.A), and an FCH-mini-PVDF flow sensor (Biotech, Germany). A three-way ball-valve then directed the sample fluid either to the hydrothermal-fluid-trap or to a 25-port valve system (McLane, U.S.A.), and then into sampling bags (MVBS, or 500 mL bags) or filters located at the back basket of the ROV Jason (Fig. 2).

#### Flushing

The Mega Pump pulled basement fluids at a flow rate of 5 L/min through the FDL. A sufficient pumping time allowed the flush of at least six times of the FDL volume to remove stagnant fluids in the FDL. Table 1 provides the flush time calculated from the pumping rate, the inner diameter of the FDL, and the lengths of the FDLs. For example, pumping at 5L/min for forty-four minutes flushed a 290 m-long FDL with 0.5-inch diameter six times. During flushing, the basement fluids also flushed the “fluid-trap” reservoir (Fig. 1).

**Table 1:** Flushing time calculated based on the inner diameters of the fluid delivery lines of a few selected CORKs installed in IODP boreholes 1362A and 1362B.

	1362B	1362A Shallow	1362A Deep
Length of Line	290	320	450
Inside Diam (in)	0.5	0.5	0.5
Inside Diam (mm)	12.7	12.7	12.7
Inside Diam (m)	0.013	0.013	0.013
Radius (m)	0.0064	0.0064	0.0064
FDL Volume (m^3^)	0.037	0.041	0.057
FDL Volume (L)	37	41	57
6 times FDL Volume (L)	220	243	342
Pumping rate (L/min)	5	5	5
Time to flush (min)	44	49	68

#### Power and communication

The MPS required a 48 V power supply from ROV Jason to the MPS controller, which operated the Mega Pump and sensors. The McLane valve system in the ROV back basket required a 24 V power supply from ROV Jason. The control van powered the MPS control box, which communicated between the MPS controller in the front basket to a custom-build computer program. The Optode oxygen sensor also received power and communication through the ROV Jason junction box and the readout shown on a screen in the control van.

#### Bag samplers

**Fig. 3:**
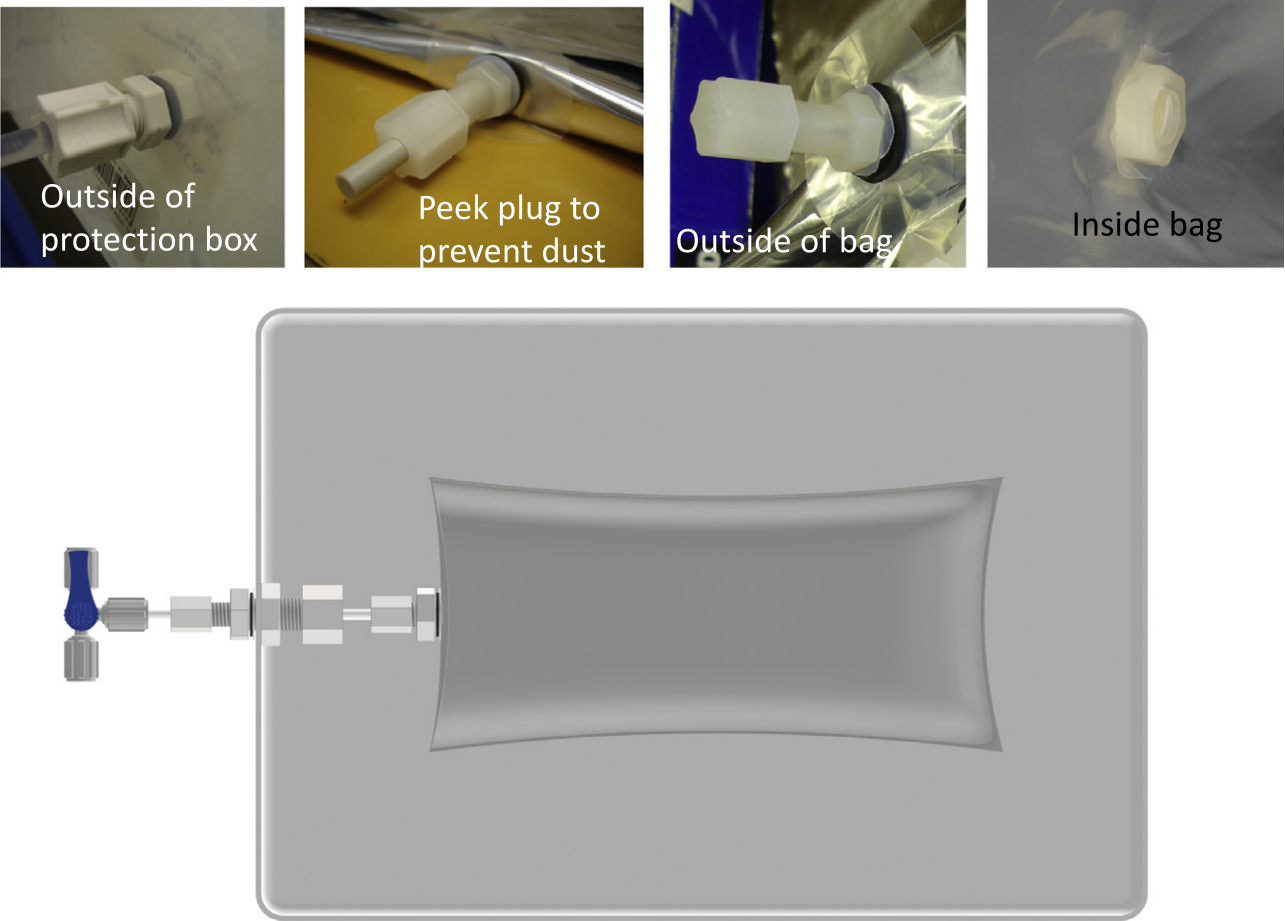
Photos and a schematic drawing of a bag sampler assemblage. An interactive 3D version of the drawing to show the bag, fittings, and box assemblage is available in the supporting material. Drawing courtesy of Fan-Chieh Chuang.

The bag samplers were composed of custom-made bags inside protection boxes (Figs. 2 and 3). The bag materials were chosen depending on the purpose of the sample fluids and the pretreatment processes [3]. For dissolved metal and dissolved organic carbon analysis, Tedlar bags (MiDan, Inc.) with PVDF valves were chosen. For molecular biology and virus research, aluminum-foil with HDPE (high density polyethylene) inside liner bags (Jensen Inert Products) were used for that the foil bags can sustain gamma irradiation (STERIS, U.S.A). The dimensions of the bags were flexible to accommodate the volume required and the shape of the protection box (Fig. S1). A series of compressional fittings, including bulkhead unions and bulkhead nuts, were used to connect the bag, the box, and to a PVDF three-way-valve. Details of the MVBS and LVBS are provided in the supporting materials (Figs. S1 and S2).

### Hydrothermal fluid-trap

**Fig. 4:**
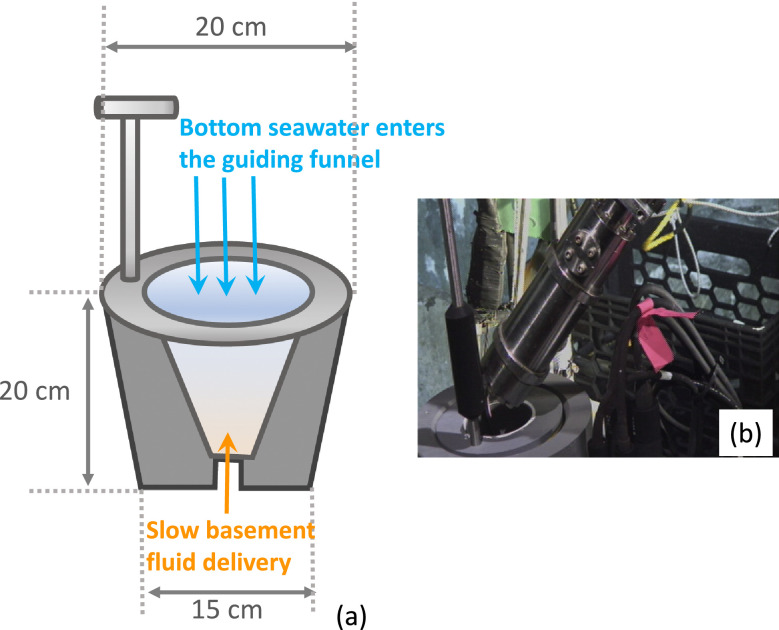
Sample funnel to guide a gastight sampler for hydrothermal fluid sampling.

**Fig. 5:**
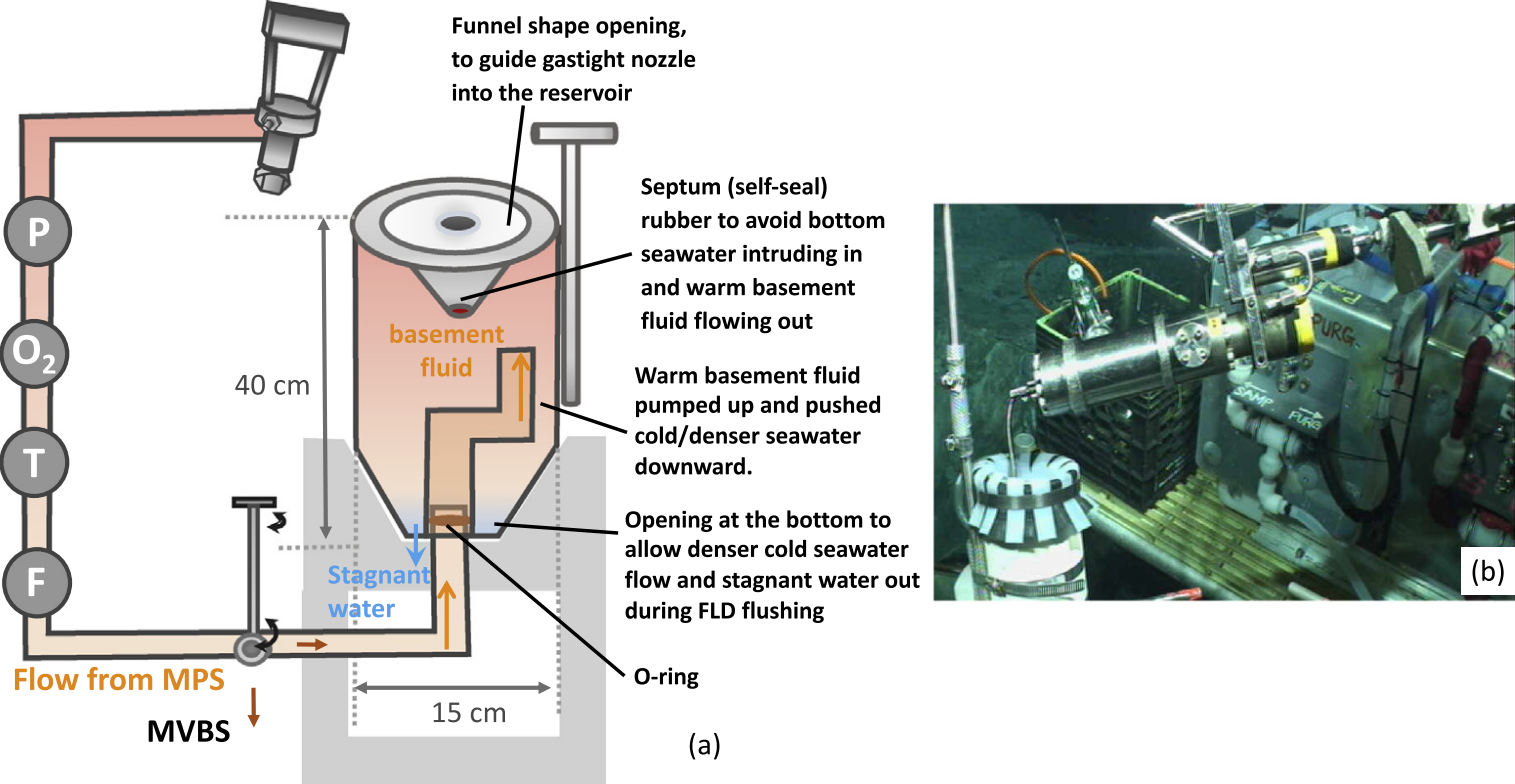
Fluid-trap version 2. (a) Schematic drawing of the trap design and the configuration on the front basket of the ROV. MVBS: medium volume bag sampler (on the back basket). P: positive displacement primary pump; O_2_: Optode oxygen sensor; T: temperature sensor; F: flow sensor. (b) Photo of the fluid-trap on the front basket.

**Fig. 6:**
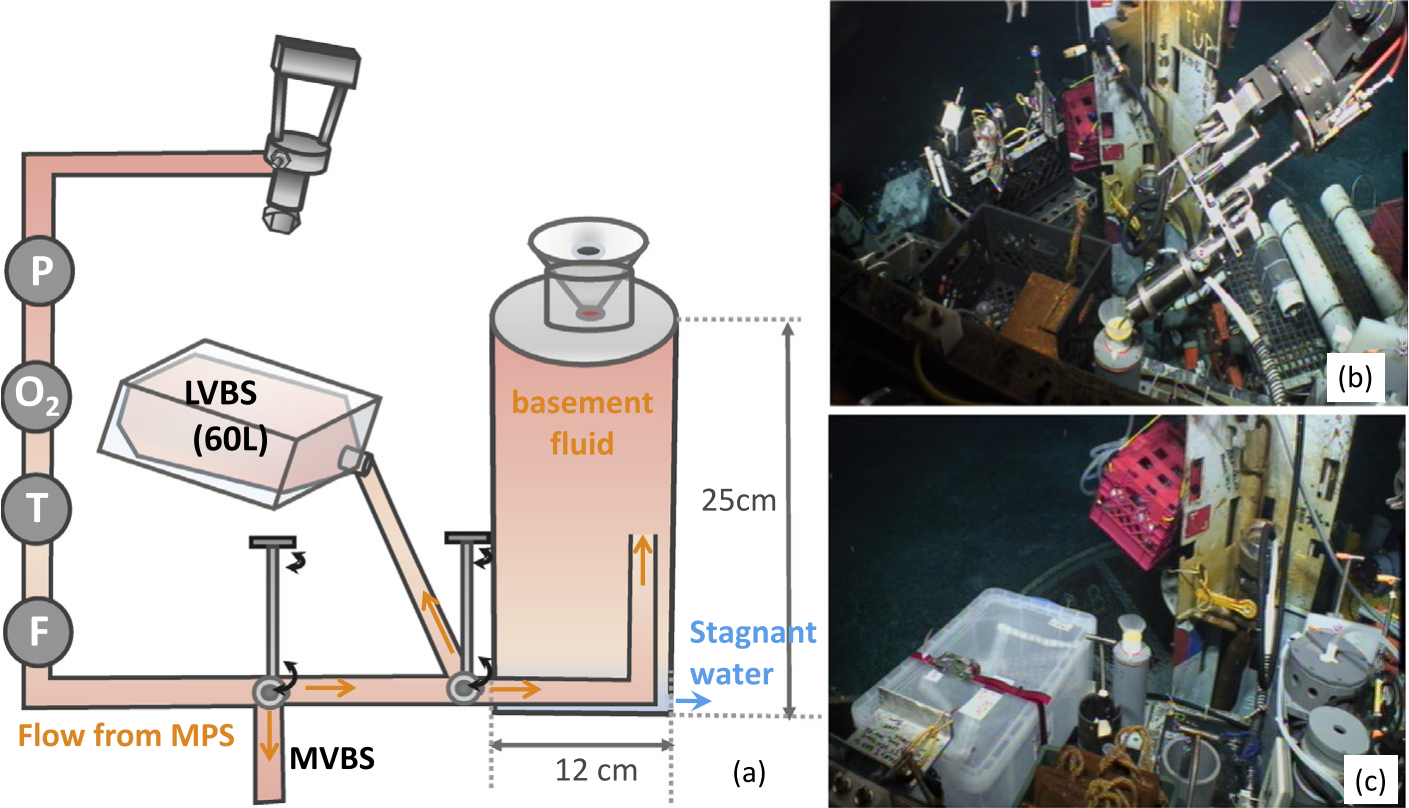
Fluid-trap version 3. (a) Schematic drawing of the hydrothermal-fluid-trap design and the configuration on the front basket of the ROV. LVBS: large volume bag sampler; MVBS: medium volume bag sampler (on the back basket). P: positive displacement primary pump; O_2_: Optode oxygen sensor; T: temperature sensor; F: flow sensor. (b) Seafloor gastight sampling photo; (c) Large Volume Bag Sampler next to the fluid-trap.

The first version of a gastight sampling reservoir in 2010 was a custom-built polyvinyl chloride (PVC) funnel that had a height of 20 cm and a largest cross-section of 20 cm (Fig. 4). Without a mechanism to prevent cold background bottom seawater from intruding into the slow-flowing hydrothermal fluid from CORK 1301A (1/8’’ FDL inner diameter), the fluid samples collected by the gastight samplers had low sample integrity [16]. The second version of the fluid-trap used in 2011 was built from a half-inch-thick (~1.2 cm) PVC pipe with a height of 40 cm and a diameter of 15 cm, holding approximately 4.5 L of fluid (Fig. 5), more than sufficient to feed a gastight (150 mL). The top of the reservoir was sealed by a 2 mm-thick rubber septum connected to the base of the guiding funnel to prevent background seawater entering the fluid reservoir. The bottom of the reservoir had an opening to a 7/8’’ Teflon tubing, with an inner diameter of 22.2 mm, that fits into the outlet of the MPS fluid pump line. The MSP outlet was a solid PVDF tubing with an outer diameter of 21.34 mm, which carried a custom-machined slot for a radial O-ring seal. The Viton O-ring (size no. 114) between the MPS outlet tubing and the trap had a cross section of 2.52 mm, an inner diameter of 15.54 mm, and an outer diameter of 20.78 mm. There was a small orifice at the bottom to expel cold seawater. The self-seal septum created a significant drag when the ROV manipulator pulled the gastight sampler out of the fluid-trap. Eventually, the funnel broke. During later expeditions, we improved the third version of the fluid-trap by securing the base onto a heavy-duty milk crate, which was bolted into the front basket of the ROV (Fig. 6). The hard-plumbing proved to be robust and remained in good condition for future usage. The third version of the fluid-trap had a height of 25 cm and a diameter of 12 cm, holding a small volume (1.8L), sufficient to provide the volume requirement by a gastight sampler.

### Switch between the fluid-trap and a large volume bag sampler (LVBS)

A three-way-vale with 1/2 NPT fitting connected to 3/8’’ PVDF tubing allowed the flowing crustal fluid to be directed to a LVBS on the front basket next to the fluid-trap (Fig. 6c). This configuration allowed a high pumping rate (5L/min) to shorten the time required to fill the 60–70L bag. The LVBS bags placed in the upright position as describe by Wommack et al. [26] have a 20% chance to be torn upon recovery. We observed that the bags were pulled toward the rigid 3/4’’ Teflon fluid inlet tubing and ripped due to the compression of air in the sampling system during sampler descending to the deep ocean. To overcome the issue, we placed the LVBS bag sideways to increase our success rate of sample recovery to 100%.

### Seafloor fixation of dissolved iron

In order to minimize contact time between crustal fluid samples and background seawater, we used the MPS system to pump basement fluid and “fix” them at the seafloor for the measurement of oxygen-sensitive ferrous iron. Two 500 mL sample bags contained 5 mL of ferrozine-acetate solution. One of the two bags also contained 5 mL of 4% ascorbic acid solution (w./v. in MQ) to reduce ferric ion (Fe^3+^) into ferrous ion (Fe^2+^). The ferrozine-acetate solution was prepared by dissolving 0.4925 g of Ferrozine in 100ml MQ and mixed with a buffer prepared by mixing 8.2 g sodium acetate with 5.7ml acetic acid in 100ml MQ. A check valve installed in the fluid line before the sample bag prevented the reagent flowing back to the 25-port valve system. Acrylic McLane remote access sampler (RAS) tubes protected the 500 mL bags. Each of the bags was programmed to collect 400 mL of basement fluid samples. Samples containing ferrous ion formed a pink iron-ferrozine complex (Fig. 2). Ferrous ion was measured directly by a Ferrozine colorimetry method [11,23] at a wavelength of 562 nm. For total dissolved iron analysis, samples were first reduced with ascorbic acid and analyzed as ferrous ion. The detection limit for both ferrous ion and total dissolved iron was 0.1 µM.

### Mobile GeoMICROBE (MGM): collection of fluid samples, filtration of suspended particles, and extraction of organic matter

**Fig. 7:**
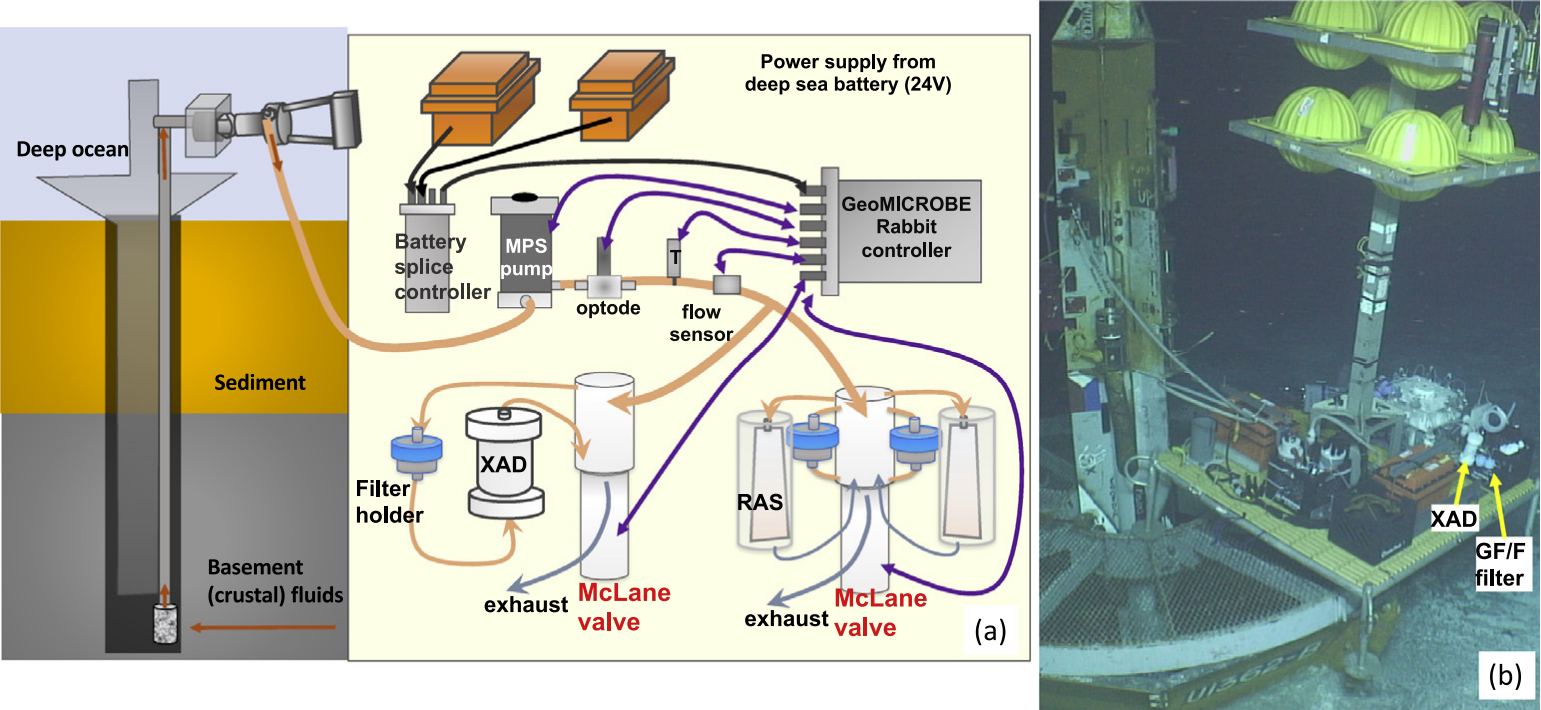
Mobile GeoMICROBE (MGM). (a) Schematic drawing of the components of the MGM to show the fluid flow, communication, and power cable configurations. The positions of each components were adjusted to maintain a balance on the deployment platform. XAD stands for the column filled with hydrophobic XAD resin that absorbs dissolved organic compounds. RAS is the remote access sampler from McLane Research Laboratories, Inc.(b) Seafloor sampling photo. The MGM utilized an ROV Jason's seafloor elevator, a platform designed to be deployed and recovered with weight and buoyancy adjustment.

The MGM (Fig. 7) is composed of a GeoMICROBE Rabbit controller, a Mega Pump, an oxygen sensor, a temperature sensor, two McLane 25-port valve manifold systems, two deep 24 V-40 A sea batteries, a battery splice controller, and an optional ODI communication system, depending on the operational requirement. The MGM is different from a standard GeoMICROBE in that the components were mounted on ROV Jason's elevator system, not on our custom-build titanium frame. The Jason elevator is made of stainless steel and is thus suitable for a few-day-term deployment, whereas the titanium GeoMICROBE frame is more suitable for a year-long deployment. The Jason elevator has a buoyance package (90 kg buoyancy) mounted directed with the platform, facilitating easy shipboard deployment and recovery. The MGM lacked the three-to-one solenoid valve system and employed only two batteries, as opposed to seven on the GeoMICROBE. The sampling time and pumping intervals of the MGM were preprogrammed with the GeoMICROBE's Rabbit control. In instances when an ODI communication system was installed, we were able to test the MGM system on the seafloor by turning on the pump and check sensor data, as well as the functionality of the two 25-port McLane valve systems, before the start of an autonomous sample collection sequence.

On the MGM, the 500 mL Tedlar bags in McLane RAS bottles, MVBS, or LVBS, were optionally configured into the 25-port valve systems to collect fluid samples. To apply MGM to filter suspended particles, we used a variety of filters, including 47mm or 25 mm pre-combusted GF/F filters, 47 mm polycarbonate filters (0.2 μm pore size), Sterivex (0.2 μm pore size). The MGM also coupled with a custom-built Teflon column packed with hydrophobic XAD-8 or Bond Elut ENV resin to extract organics for characterization. The feed sample to the absorbent resin had passed pre-combusted 47 mm GFF filter, i.e., to obtain the dissolved organic fraction. A McLane pump instead of the Mega pump controlled the extraction flow rate of 50-125 mL/min to ensure a minimum contact time of 2 min between the hydrothermal fluid and the resin, provided a column size is of 125–500 mL (the large version shown in Fig. 7b). While the MVBS and LVBS have their custom-built frames, the RAS bottles, filters, and the columns were secured either on a custom-built polyethylene (PE) rack or on a heavy-duty milk crate mounted onto Jason's elevator. Upon recovery of the filters and extraction columns, the residual fluids in the filter holders, columns, or in the tubing were collected and analyzed for their Mg concentrations to ensure sample quality (e.g., [16]). After sample recovery, the XAD column was stored at -20°C until thawed for elution in a shore-based laboratory. After thawed, the column was first rinsed with three times volume size of Milli-Q water to remove salt, and then the absorbed organic compound was eluted with HPLC grade methanol, rotary evaporated dried, freeze-dried, then redissolved in 50 mL ND_4_OD for characterization.

## Method validation

**Fig. 8:**
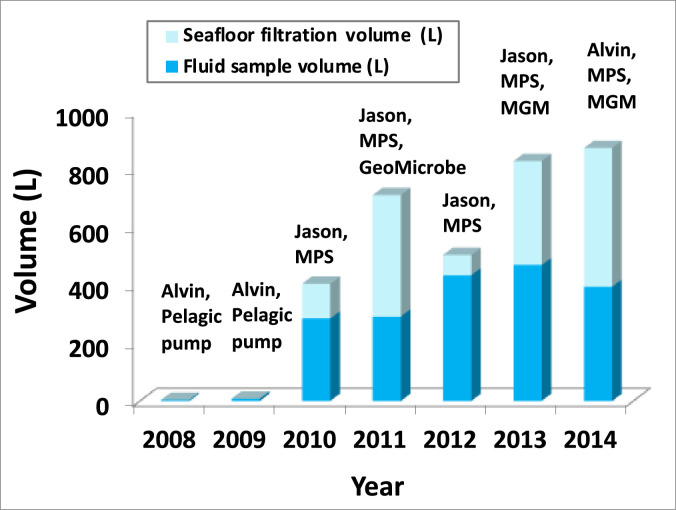
Basement fluid sample inventory based on the samples recovered during cruises AT15-35 (2008), AT15-51 (2009), AT15-66 (2010), AT18-07 (2011), AT26-03 (2013), and AT26-18 (2014) to the CORK clusters located on the eastern flank of the Juan de Fuca Ridges and during cruise MSM20-5 (2012) to the North Pond. Submersible Alvin or remotely-operated-vehicle ROV Jason were used to assist seafloor operation. Sampling methods included pumping with a pelagic pump, mobile pumping system (MPS), advanced instrumented GeoMICROBE sled, mini GeoMICROBE (MGM).

Throughout seven research cruises, the utilization of MPS and MGM has significantly improved the sample fluid volume (Fig. 8). In our early cruises, we utilized a pelagic pump but the pump was not powerful enough to overcome the drag of the small inner diameter (1/8’’) of 1301A FDL. Approximately only 4-8 L high quality basement fluid samples were collected. After the application of the MPS, our sampling capacity greatly increased to 280-470 L of basement fluids and between 70-120L seafloor filtration (year 2010 & 2012). The deployment of a GeoMICROBE Sled or a MGM system greatly enhanced the seafloor filtration capacity up to 480 L. The samples have been used to study primordial gas [18], dissolved methane and hydrogen [17], dissolved organic carbon (e.g., [19,24]), molecular microbiology (e.g., [2,^13^,14]), and viruses [22] of the deep biosphere.

Here, we focus on the results of the seafloor fixation experiment (Table 2; Fig. 2b). The ferrous ion and the total dissolved iron in the basement fluid samples were fixed with Ferrozine once collected into the 500 mL bag, leaving little time for the ferrous ion to react with oxygen that was initially in the bag. Oxidation turns ferrous ion into ferric ion or iron oxyhydroxide precipitates. In the reducing basement environment, the ferrous ion is the dominate dissolved iron species, and the ferrous/total dissolved iron ratios were near 100% in the two seafloor fixation samples. The total dissolved iron and the ferrous ion concentrations in the corresponding MVBS samples decreased dramatically to less than 7%, indicating that iron precipitation and ferrous ion oxidation had occurred between the time of fluid collection and sample analysis. Thus, we suggest conducting seafloor fixation for research that requires precise and accurate quantification of reducing species. The versatile design of the MPS and the MGM allows seafloor fixation.

**Table 2:** Seafloor fixation experiment for ferrous ion and total iron measurement; cruise # AT18-07.

Sample site	Sample bag	Treatment	Total dissolved Fe (TdFe, μM)	Fe^2+^ (μM)	Fe^2+^/TdFe (%)
	**MPS**				
1362A	500 mL bag	seafloor fixation	1.6	1.6	100
1362B	500 mL bag	seafloor fixation	1.5	1.4	95
1362A	MVBS	-	0.6	0.04	7
1362B	MVBS	-	1.0	0.00	0

**Fig. 9:**
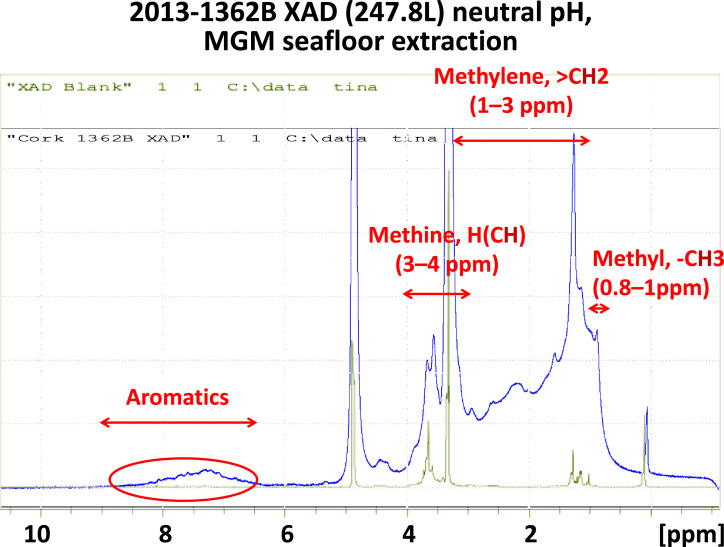
NMR spectra of the XAD extracted hydrophobic dissolved organic compounds for basement fluid samples (blue line) and the XAD resin blank (deployed but not sampled) to investigate the functional group compositions. The red circle marks the proton chemical shifts between 5.3 and 8.3 ppm, which is indicative of aromatic compounds.

We demonstrated the characterization of a XAD extracted dissolved organic matter with nuclear magnetic resonance (NMR) spectrometry (Fig. 9) of a sample collected with the MGM (Fig. 7). The XAD resin extracted a significant amount of hydrophobic dissolved organic matter from a total of 247 L of basement fluid samples. The NMR spectrum showed a continuous broad peak from 0.8 to 5.0 ppm, with a few pronounced peaks at 1.3, 3.3, and 3.5 ppm, which are regions typically assigned as the hydrogen-bonded to methyl, methylene, methine, respectively. The pronounced elevated broad peaks between 5.3 and 8.3 ppm is indicative of aromatic compounds, which have also been described with basement fluid samples collescted with MVBS and LVBS and extracted shipboard with Bond Elut PPL resins [19]. While the NMR is a non-destructive analysis, the remaining samples can be used for fluorescence compound and other characterization analysis.

## Future applications

### Seafloor incubation experiments

The MGM can be applied to initiate incubation experiments by mixing stable isotope labels (e.g., ^13^C-HCO_3_^−^,^15^N-NH_4_^+^, and ^15^N-NO_3_^−^) with basement fluid samples collected in the 500 mL Tedlar bags. A seafloor incubation is particularly plausible for a low-temperature system such as the North Pond basement fluids (e.g., 10°C) whereas the heating components may take too much power to maintain at an in situ hydrothermal temperature (e.g., 65°C) for the warm ridge flank sites such as the eastern flank of the Juan de Fuca Ridge. The seafloor incubation can also include deep seawater samples as control.

### Hydrothermal vent fluid sample from other geological settings

The MPS and MGM can collect fluid, particles, and extract organics from diffuse vent fluids with low-particle and relatively low-gas content. An inverted funnel, instead of the Jannasch connector, acts as the fluid intake port. To use the MPS and MGM for collecting samples from vent fluids with high particle contents, a large diameter, and large pore size (e.g., 2.0 µm pore size, hydrophilic glass fiber, 142 mm diameter) filter need to be installed. Otherwise, clogging inside the pump and the 25-port-valve system may occur.

## Declaration of Competing Interest

The authors declare that they have no known competing financial interests or personal relationships that could have appeared to influence the work reported in this paper.

## Appendix Supplementary materials

Image, application 1

Image, application 2
