# Supplementary material for: Sampling of basement fluids via Circulation Obviation Retrofit Kits (CORKs) for dissolved gases, fluid fixation at the seafloor, and the characterization of organic carbon
Source: MethodsX. 2020 Aug 15;7:101033. doi: 10.1016/j.mex.2020.101033 (PMC7482021; doi:10.1016/j.mex.2020.101033)
Supplement: Supplementary file 1 [file mmc1.pdf]

Not draw to scale.

TITLE	Bag_sampler_3D_plot
PART NO	Schematic Drawing
REVISION	1.0
DESIGNEF	Fan-Chieh Chuang
ENGINEEF	Chih-Chiang Hsieh

NOTES

Not draw to scale.

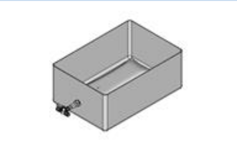

View

THE INFORMATION AND/OR MATERIAL IN THIS DOCUMENT IS THE PROPERTY OF AND RESTRICTED INFORMATION AND/OR MATERIAL OF THE AUTHOR. THIS INFORMATION MAY NOT BE USED, REPRODUCED, PUBLISHED OR DISCLOSED TO OTHERS WITHOUT WRITTEN AUTHORIZATION. IT IS TO BE USED ONLY FOR MANUFACTURING ITEMS SPECIFIED WITHIN THE DOCUMENT.
