# Supplementary material for: Sampling of basement fluids via Circulation Obviation Retrofit Kits (CORKs) for dissolved gases, fluid fixation at the seafloor, and the characterization of organic carbon
Source: MethodsX. 2020 Aug 15;7:101033. doi: 10.1016/j.mex.2020.101033 (PMC7482021; doi:10.1016/j.mex.2020.101033)

Open the file.
Enable 3D content by clicking the options. Select trust this document.


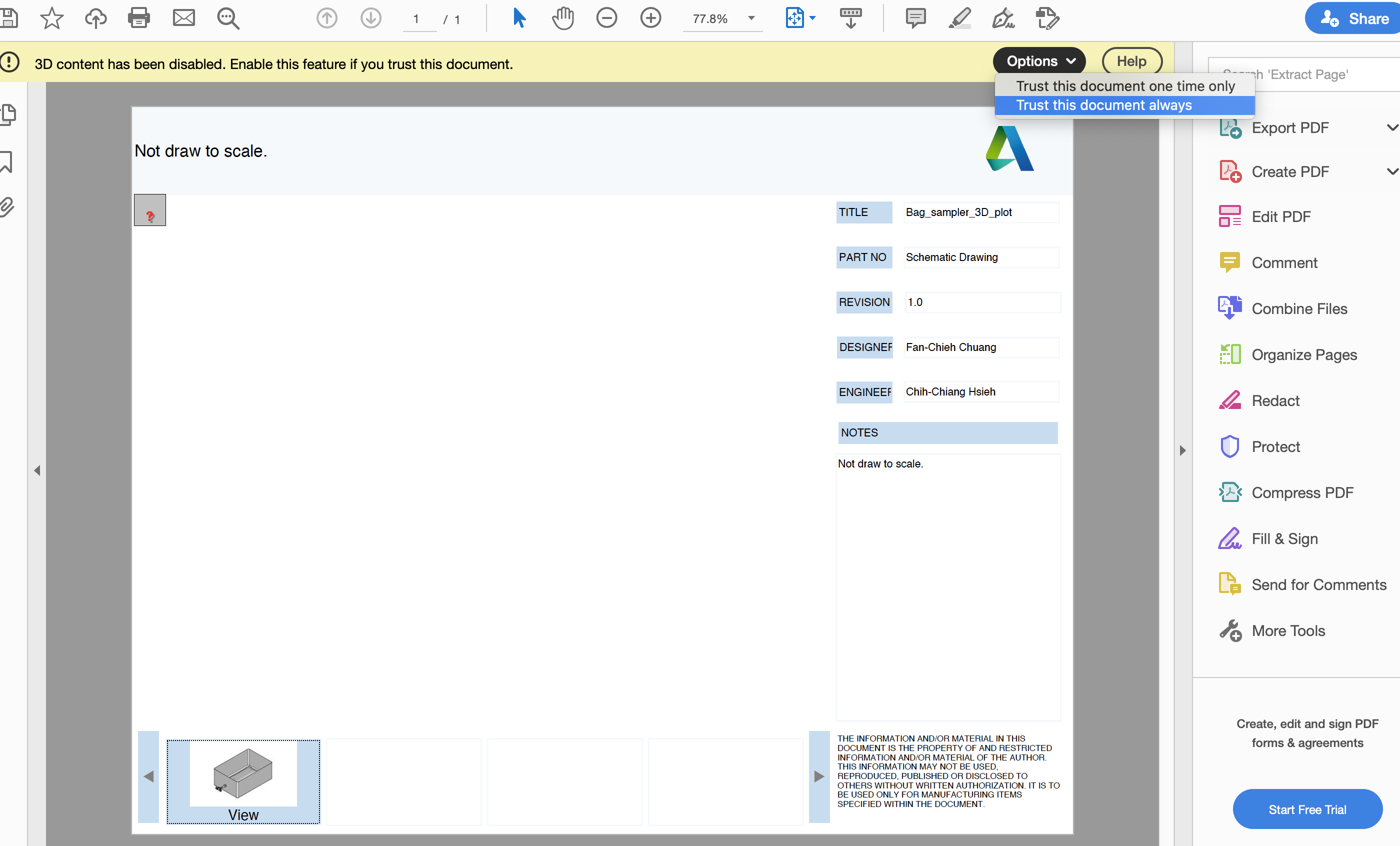


Double click on the small image at the bottom left. The drawing will display in the view window.


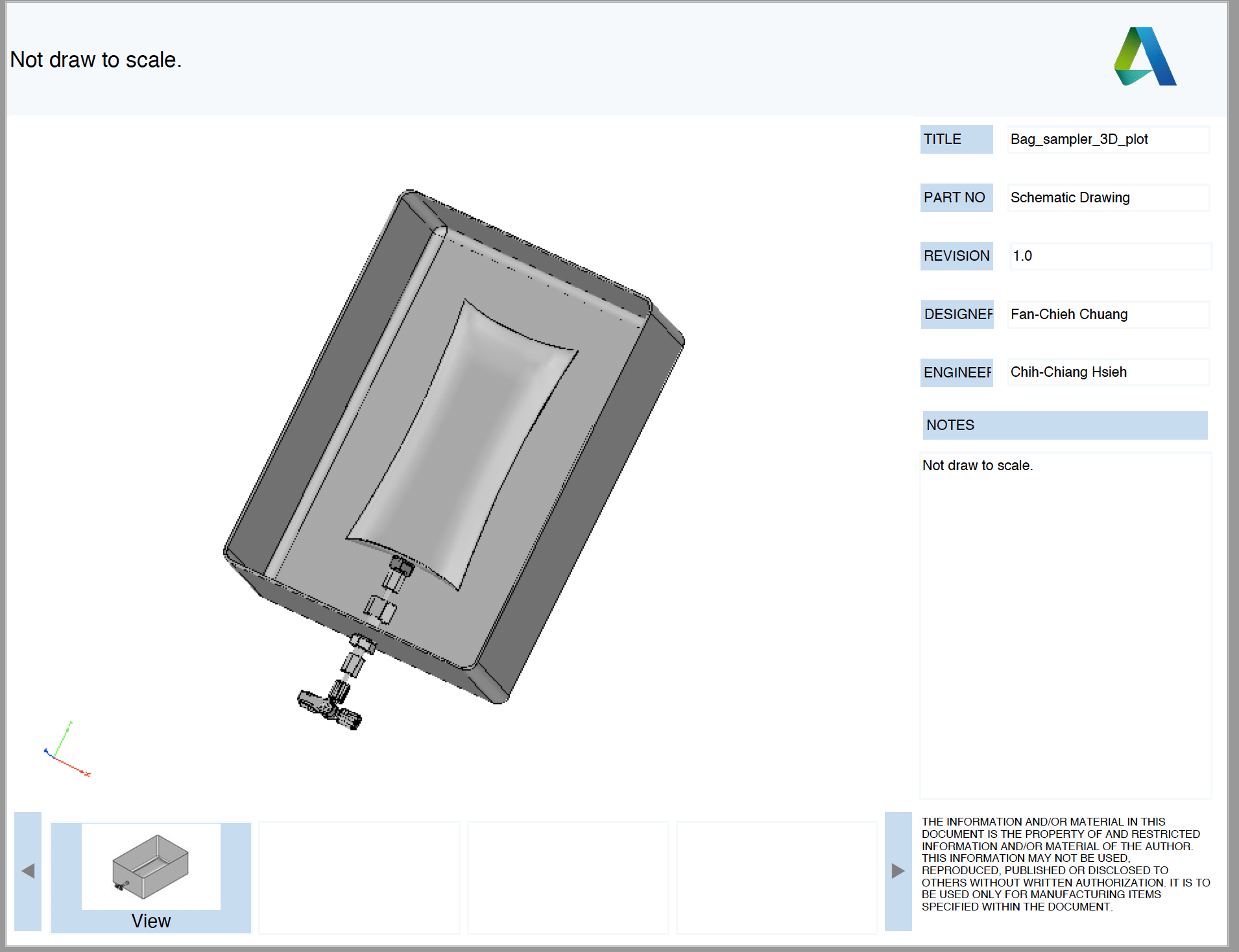


Rotate or enlarge to see the details. Please note that the fittings, the bag, or the box are not drawn to scale. It is for easy visualization of our bag sampler assemblage.


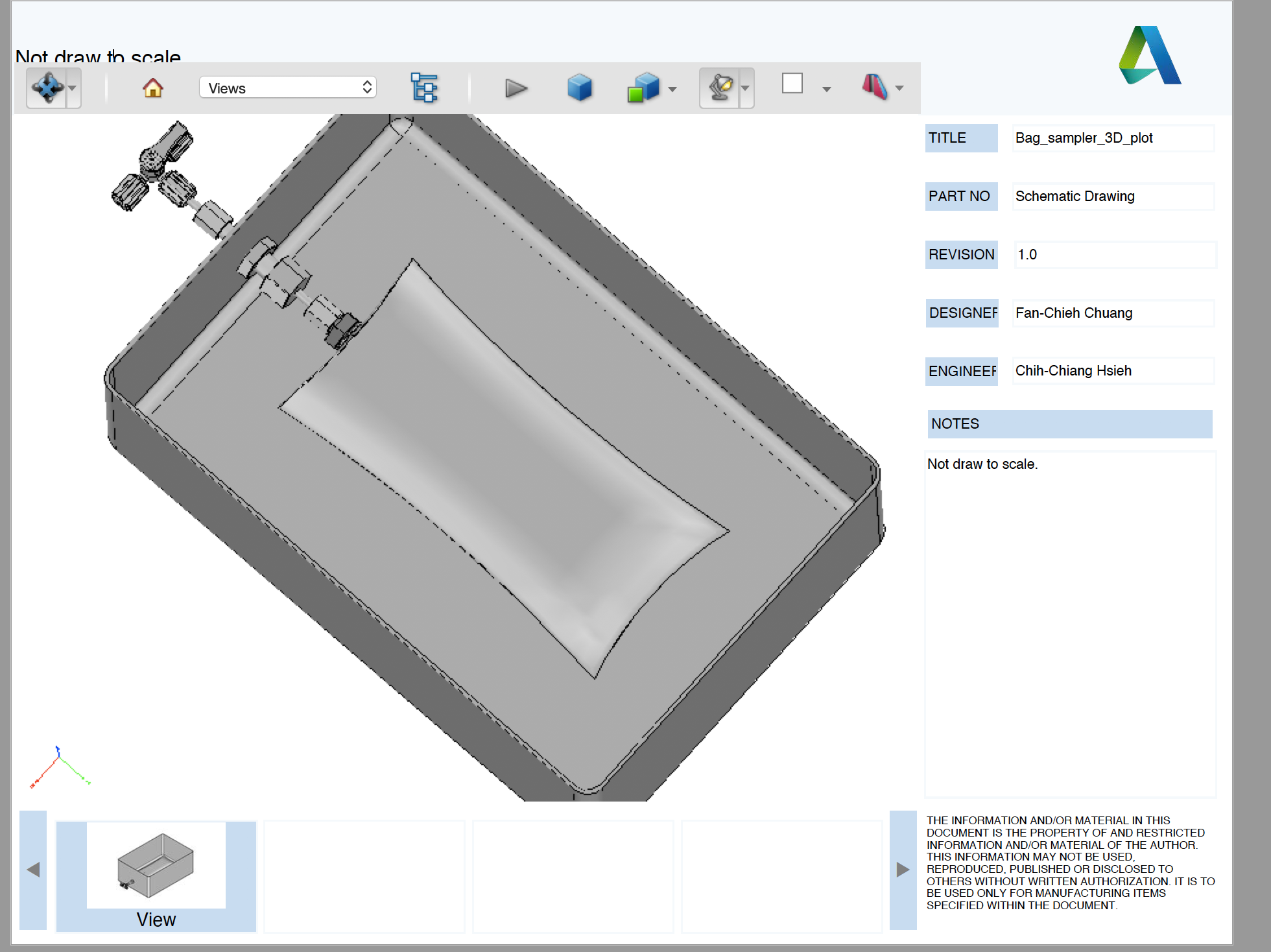

Supplement: Supplementary file 2 [file mmc2.docx]
